# Relationship between rural self-efficacy and rural career intent after rural clinical training: a study on medical students in Japan

**DOI:** 10.1186/s12909-022-03511-7

**Published:** 2022-06-08

**Authors:** Ryuichi Kawamoto, Daisuke Ninomiya, Asuka Kikuchi, Yoshio Tokumoto, Teru Kumagi

**Affiliations:** 1 Department of Community Medicine, Ehime University Graduate School of Medicine, Toon-city, Ehime 791-0295 Japan; 2 Department of Internal Medicine, Seiyo Municipal Nomura Hospital, 9-53 Nomura, Nomura-cho, Seiyo-city, Ehime, 797-1212 Japan

**Keywords:** Rural self-efficacy, Medical student, Rural career intent, Cohort study

## Abstract

**Background:**

In Japan, community medicine clerkships facilitate positive attitudes toward rural medical practice and encourage rural recruitment. Rural self-efficacy has been shown to influence rural career intent following a rural clinical placement. However, the impact of subjective difficulties of living in a rural area on future rural career intent is also important. This study aims to explore whether rural self-efficacy influences the relationship between difficulty with living in a rural area and rural career intent.

**Methods:**

The subjects included 308 male and 255 female participants aged 20–41 [median (interquartile range): 22 (21–22)] years. Rural self-efficacy was based on a validated scale consisting of 15 questions. Difficulty with living in a rural area was measured asking students. A cohort survey was conducted to evaluate the effect of the rural self-efficacy score on the rural career intent of Japanese medical students after they completed their rural clinical training.

**Results:**

The following variables were significantly associated with a higher rural self-efficacy score: female sex (*p* = 0.003), age < 21 years (*p* = 0.013), having a doctor as a role model (*p* < 0.001), gaining admission through a school recommendation (*p* = 0.016), living in a rural or remote area until the age of 18 years (*p* = 0.018), and orientation towards general medicine (*p* < 0.001). In addition, baseline difficulty with living in a rural area was significantly associated with a lower self-efficacy score (*p* < 0.001). Participants with a stronger intent to practice in a rural area before rural clinical training had higher rural self-efficacy and showed a stronger positive rural career intent after rural clinical training (*p* < 0.001). A multivariable logistic regression analysis demonstrated that difficulty with living in a rural area [odds ratio (OR): 0.61; 95% confidence interval (CI), 0.39–0.84] was still associated with lower rural career intent after rural clinical training, independent of all confounders such as gender, age, scholarship for regional duty, rural background, and orientation towards general medicine. However, when rural self-efficacy (OR, 1.12; 95% CI, 1.07–1.16) was added as a factor for rural career intent, difficulty with living in a rural area (OR, 0.68; 95% CI, 0.43–1.06) was no longer observed as an associated factor.

**Conclusion:**

Subjective difficulty with living in a rural area was shown to reduce future rural career intent, but high rural self-efficacy ameliorated this decline.

## Background

The geographic maldistribution of physicians and consequent shortage of physicians in rural areas is a serious social issue in Japan as well as the rest of the world [1, 2]. The factors that affect the intent of medical students to engage in rural careers have been widely investigated. These factors include gender [3], rural background [4–6], receiving scholarships for regional duty [7], early exposure to the community during medical training [4], high academic performance upon entry [8], rural clinical training environments [3–6, 9, 10], and professional specialties (e.g., general medicine) [6]. However, few studies applied psychosocial constructs, which have been well-established in career choice models, to the development of rural medical careers [6, 11].

The subjective difficulties associated with living in a rural area may include perceived life inexperience in the rural setting, which may correspond to life stage (e.g., having partners and/or children). A number of factors contribute to these difficulties, such as social isolation, lack of cultural diversity, lack of accessibility, lack of quality educational structures (kindergartens/schools), lack of accommodation, poor internet access, lack of transport, and lack of financial support [12–14]. Subjective difficulties with living in a rural area were found to produce a negative effect on rural career intent among medical students [12, 13].

Bandura’s self-efficacy theory [15], which is derived from the social cognitive model of behavior, has received much attention in the literature [16], and scholars have examined self-efficacy by drawing on Bandura’s four sources of self-efficacy: mastery experiences, vicarious experiences, verbal persuasion, and emotional and physiological reactions [17]. Self-efficacy is defined as “people’s beliefs in their capabilities to produce designated levels of performance” [17, 18]. Self-efficacy in rural practice has recently emerged as a factor for the intent to pursue a future rural career [19, 20], and reflects medical students’ beliefs and expectations that they may be able to become successful rural physicians in the future [6, 11, 12]. Our previous cross-sectional studies have reported a strong association between rural self-efficacy and intent to practice medicine rurally [21]. One study has shown that the opportunity for experience increasing autonomy through longitudinal integrated clerkships (i.e., mastery experiences of bandura) enhances students’ self-efficacy for rural practice [22]. A higher level of self-efficacy for rural practice could diminish effects of perceived social isolation associated with living in a rural area [12, 23]. However, career-based psychosocial motivations to practice medicine in rural areas need to be better understood, it is unclear whether earlier educational work experiences enhance rural career intent by rural self-efficacy.

Thus, this study was designed as a self-administered cohort survey for Japanese medical students to evaluate subjective difficulties with living in a rural area and the effect of rural self-efficacy on rural career intent after rural clinical training.

## Methods

### Participants

The study was designed as a cohort study. We conducted a survey of 5^th^ year medical students (approximately 100 each year, for a total of approximately 800 students) from one Japanese regional university school of medicine were surveyed in each year from 2013 to 2020. They were asked to complete all of the questionnaires using a written questionnaire during orientation within 4 weeks before clinical practice in a rural area, and it via email after completion of clinical practice in a rural area. Individual responses were anonymous, and questionnaire completion was voluntary. The study was approved by the ethics committee of Ehime University (Institutional Review Board: 15,070,004). Informed consent was obtained from all subjects.

### Questionnaire

This tool has been previously described and validated [21]. The questions were based on four sources of self-efficacy: work preferences, evaluation of rural practice, rural living preferences, and personal character, and 15 questions were designed to measure self-efficacy in rural medical practice (Table 1). In addition, these questions also include self-assessments of unique work preferences and personal characteristics that are essential to Japanese healthcare in rural areas, which previous studies have not included [19, 20, 24]. The questions were scored based on a Likert scale, and a composite score of rural medical self-efficacy was calculated. This score was obtained by summing up the scores of all items (i.e., lowest score is 15 and highest score is 60). Cronbach’s α, indicating internal reliability, was 0.849 for the current sample [21]. In summary, the survey contains the questions required to assess self-efficacy, as described by Bandura [15], and has been adapted for the assessment of rural medical careers.

**Table 1:** Survey questions aligned with four sources of rural self-efficacy

Sources of rural self-efficacy
**Questions** **strongly agree = 4 / slightly agree = 3 / slightly disagree = 2 / strongly disagree = 1**
Factor 1: Work preferences
I would like to be concerned with a patient's life throughout treatment
I would also like to support the patient’s welfare
I want to be a doctor who walks with the patient and works with the patient on their problems
I would like to provide continuous care for the patient from an early stage
I am interested in the patients themselves (e.g., children and older adults)
Factor 2: Evaluation of rural practice
There are many opportunities in rural areas that can improve one’s career
Working in a rural area provides more opportunities to practice a variety of skills
There will be opportunities for community medicine in rural areas
I am interested in research activities in the rural field
Rural practice provides greater opportunities for work autonomy
Factor 3: Rural living preferences
Living in rural areas does not bother me
I would like to bring up a child in a rural area
There are things I enjoy doing in rural areas
Factor 4: Personal character
I like to talk with people
I like to talk with medical colleagues (e.g., nurses)

The score is obtained by *summing up the scores* of *all* items, with the lowest possible score being 15 and the highest possible score being 60

The questionnaire probed the participant’s background and intent to engage in rural practice. The questions addressed several socio-demographic factors, including gender and age. Participants were asked if they gained admission while they were living in their hometown, if they had graduated from a public high school, graduated from combined junior high and high school, failed the entrance exam, had parents who were doctors, had a doctor as a role model, received a scholarship for regional duty (chiiki-waku), acquired admission through a school recommendation, and had experience with admission to another university. The size of the hometown where participants lived until they were 18 years (i.e., a rural or remote area; town or village, population of 10,000 to 50,000; a small city, population of 50,000 to 100,000; a medium-sized city, population of 100,000 to 500,000; or a large city, population of ≥ 500,000), and their orientation towards general medicine were also queried. Furthermore, participants were asked subjectively if they thought that living in a rural area was difficult (i.e., strongly agree, agree, disagree, or strongly disagree), and about their intent to practice in a rural area (i.e., actively willing to work, willing to work for a certain period, avoid as much as possible, absolute refusal, or other). Their degree of intent to practice in a rural area was divided into two groups: those who actively willing to work and those who were willing to work for a certain period, and those who wished to avoid working rurally as much as possible, those who absolutely refused, and others. Participants who completed their clinical training in rural areas were asked to state the type of region where they wanted to continue their practice (i.e., an urban, it’s more of an urban area, it’s more of a rural area, or a rural area as rural group). The former two groups were combined into “urban” and the latter two groups were combined into “rural” for analysis.

### Statistical analyses

Statistical analyses were conducted using IBM SPSS Statistics version 25 (Statistical Package for Social Science, Chicago, IL, USA). Data are presented as mean ± standard deviation (SD), unless specified otherwise and for parameters with non-normal distribution (such as age), the data are shown as median (interquartile range) values. Student’s t-test, and an analysis of variance (ANOVA) with the Bonferroni correction for multi-comparisons in which there were more than three sub-groups were also performed in order to characterize the relationships between baseline characteristics and rural self-efficacy. McNemar's test were also performed to compare the intent to engage in rural practice before and after rural clinical training. Finally, a logistic regression analysis was conducted to evaluate the contribution of each confounding factor for rural career intent (e.g., “a rural area” versus “an urban area”). Model A was adjusted for all variables except for rural self-efficacy, and Model B was adjusted for all variables including rural self-efficacy. A value of *p* < 0.05 was considered significant.

## Results

### Participant characteristics

Table 2 presents the characteristics of participating medical students. The study sample comprised 563 students (a response rate of 60.3%), of whom 308 were male and 255 were female. Participants’ age ranged between 20 and 41 [median (interquartile range), 22 (21-22)] years. An estimated 49.7% gained admission from their hometown, 8.3% had been admitted to another university, 27.9% had a parent who was a doctor, and 40.7% had a doctor as a role model. Further, 21.7% received a scholarship for their regional duty and 28.8% gained admission with help from a school recommendation. About 11.9% of the medical students reported having a rural background and 18.8% were oriented toward general medicine.

**Table 2:** Baseline characteristics of medical students

Baseline characteristics *N* = 563	N (%)
Gender	
Female	255 (45.3)
Male	308 (54.7)
Age, median (interquartile range): 22 (21–22) years	
< 21 years	257 (45.6)
≥ 21 years	306 (54.4)
Admission while living in hometown	
Yes	280 (49.7)
No	283 (50.3)
Graduation from public high school	
Yes	276 (49.0)
No	287 (51.0)
Graduation from junior high and high school	
Yes	297 (52.8)
No	266 (47.2)
Has failed the entrance exam	
Yes	250 (44.4)
No	313 (55.6)
Work experience	
Yes	25 (4.4)
No	538 (95.6)
Experience with admission to another university	
Yes	47 (8.3)
No	516 (91.7)
Had a parent who is a doctor	
Yes	157 (27.9)
No	406 (72.1)
Had a doctor as a role model	
Yes	229 (40.7)
No	334 (59.3)
Scholarship for regional duty	
Yes	122 (21.7)
No	441 (78.3)
Admission by school recommendation	
Yes	162 (28.8)
No	401 (71.2)
Hometown of residence size until 18 years of age	
Rural, remote, town, or village	67 (11.9)
Small or middle city (i.e., population of 50,000 to 500,000)	374 (65.4)
Large city (i.e., population of ≥ 500,000)	122 (21.7)
General medicine oriented	
Yes	106 (18.8)
No	457 (81.2)

### Baseline factors associated with rural self-efficacy before rural clinical training

Table 3 presents the results for the relationship between participants’ baseline characteristics and rural self-efficacy before rural clinical training. The following variables were significantly associated with a higher rural self-efficacy score: female sex (*p* = 0.003), age < 21 years (*p* = 0.013), having a doctor as a role model (*p* < 0.001), gaining admission through a school recommendation (*p* = 0.016), and orientation towards general medicine (*p* < 0.001). The rural self-efficacy score was significantly higher in those residing in rural or remote areas until 18 years of age as opposed to those who lived in a large city until 18 years of age (*p* = 0.018). No other variable was significantly associated with the self-efficacy score.

**Table 3:**
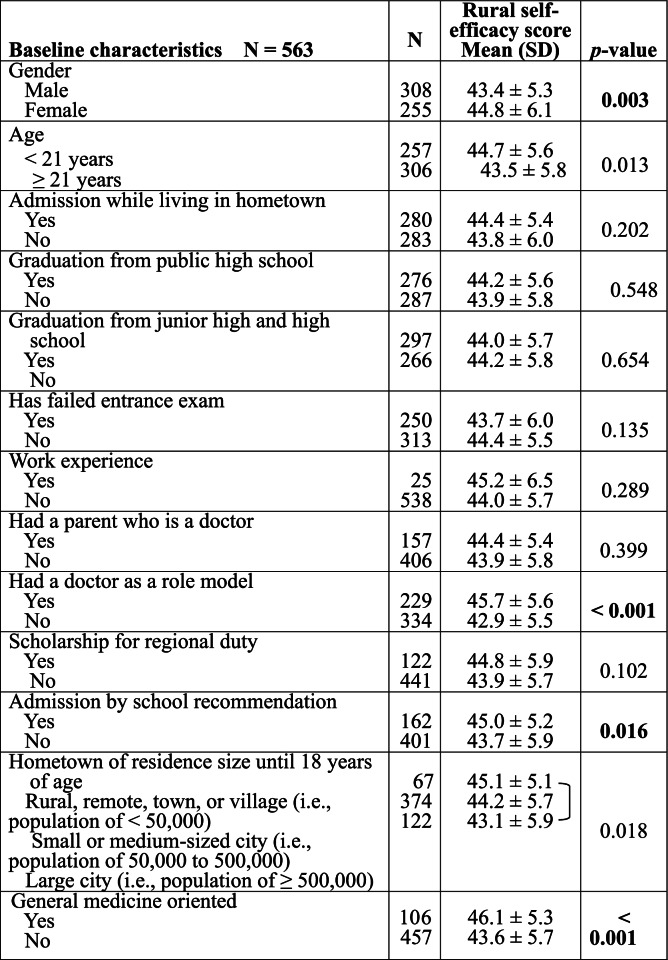
Relationship between baseline characteristics and rural self-efficacy before rural clinical training

Data are presented as mean ± standard deviation (SD). Bold indicates significance (*p* < 0.05)

### Relationship between baseline difficulty with living in a rural area and rural self-efficacy before rural clinical training

As shown in Table 4, baseline difficulty with living in a rural area was significantly associated with a lower self-efficacy score, and those with positive opinions had significantly lower rural self-efficacy scores than those with negative opinions (*p* < 0.001).

**Table 4:** Relationship between baseline difficulty with living in a rural area and rural self-efficacy before rural clinical training

Baseline characteristics *N* = 563	N	Rural self-efficacy score Mean (SD)	*p-value*
Difficulty with living in a rural area			
Strongly agree	27	39.4 ± 6.1^a,b,c^	** < 0.001**
Agree	197	43.4 ± 6.0^a^	
Disagree	295	44.5 ± 5.1	
Strongly disagree	44	46.8 ± 6.5	

Data are presented as mean ± standard deviation (SD)

^a^*p* = 0.001 versus “strongly disagree”,

^b^*p* = 0.001 versus “disagree”,

^c^*p* = 0.003 versus “agree”

### Relationship between intent to practice in a rural area and rural self-efficacy score before rural clinical training

**Fig. 1:**
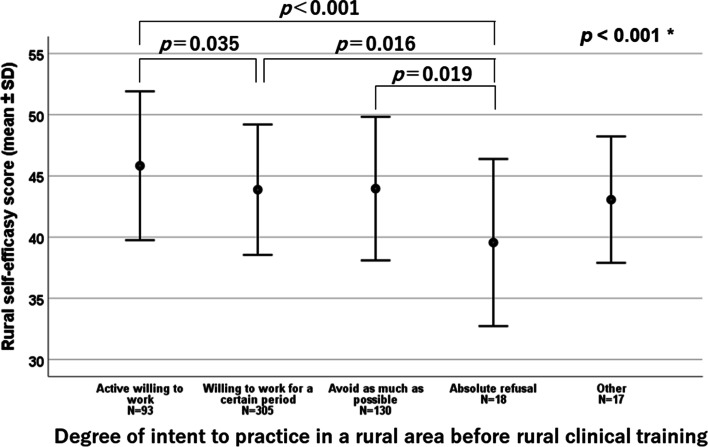
Relationship between the intent to engage in rural practice and the rural self-efficacy score before rural clinical training. Among the responses for the “Other” category, “don’t know, depends on the conditions” was the most common response

Figure 1 illustrates the relationship between subjects’ intent to practice in a rural area and the rural self-efficacy score before rural clinical training. The rural self-efficacy score significantly increased with a stronger degree of intent to practice in a rural area (*p* < 0.001).

### Rural practice intent before and after rural clinical training

**Fig. 2:**
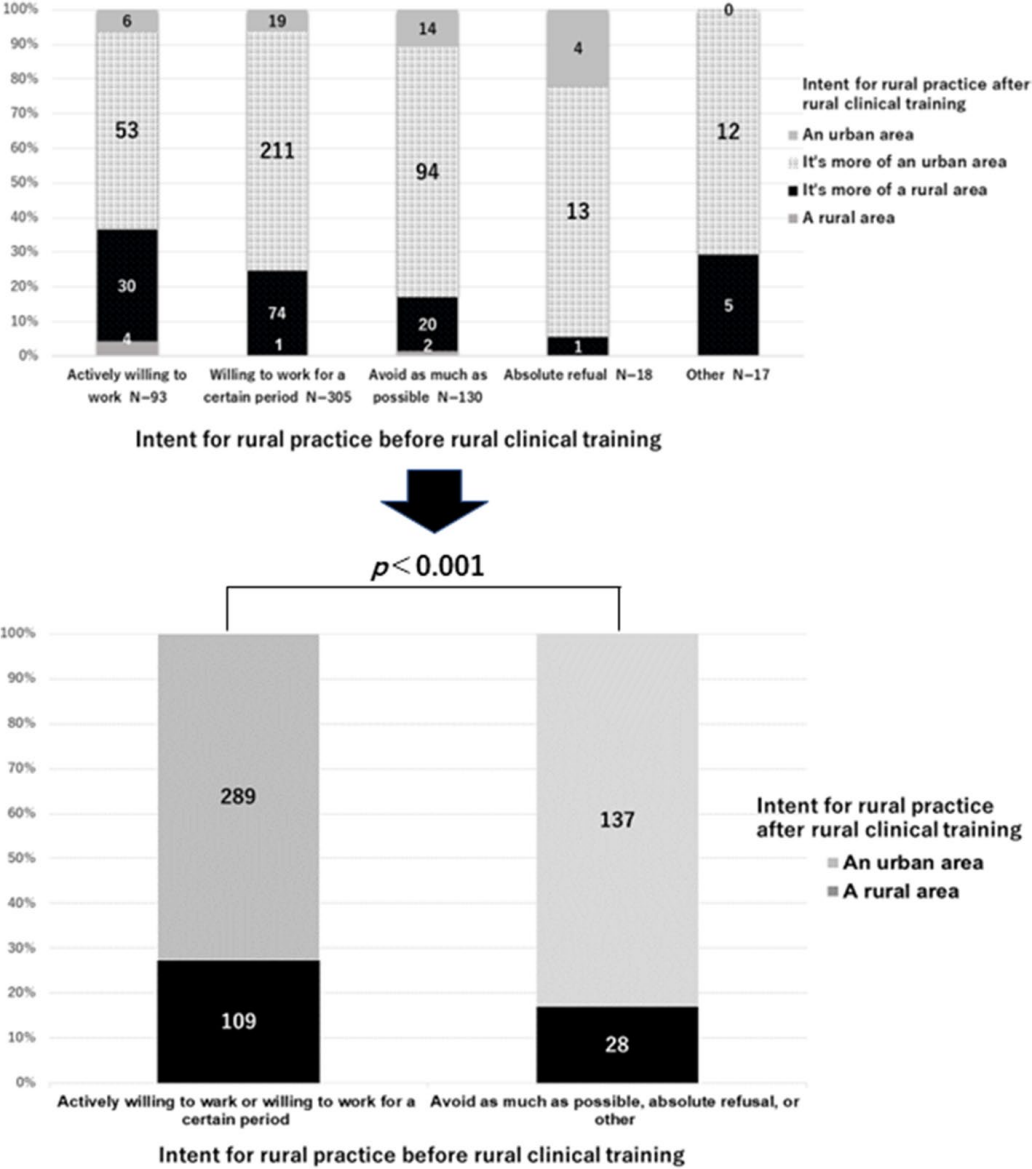
Intent to engage in rural practice before and after rural clinical training. The lower figure shows the results of dividing the upper figure into two groups with respect to the intent to engage in rural practice before and after rural clinical training

Figure 2 shows the results for their intent to practice in a rural area before and after rural clinical training. As shown in the upper panel of the figure, the stronger the intent for rural practice in a rural area before rural clinical training, the stronger the intent to practice in a rural area after training, excluding "other". Furthermore, in the lower panel of the figure, the relationship between positive and negative intent for rural practice before and after rural clinical training was statistically examined, and a positive group before training was significantly associated with positive rural career intent after training (*p* < 0.001).

### Logistic regression analysis of baseline characteristics including effect of rural self-efficacy on rural career intent after rural clinical training

Table 5 displays the results for the simple (non-adjusted) and multivariable logistic regression analyses. The non-adjusted model showed that the following factors increased participants’ intent to engage in a rural practice after rural clinical training: scholarship for regional duty [odds ratio (OR): 2.01; 95% confidence interval (CI); 1.30–3.11], rural background (OR: 2.74, 95% CI, 1.62–4.65), orientation towards general medicine (OR: 2.25, 95% CI, 1.43–3.54), active intent to practice in a rural area before rural clinical training (OR: 1.85, 95% CI, 1.16–2.93), and higher rural self-efficacy (OR: 1.12, 95% CI, 1.08–1.16). In Model A, multivariate adjustment was made for all factors except rural self-efficacy, and in Model B, multivariate adjustment was made for all factors including rural self-efficacy. Model A demonstrated that difficulty with living in a rural area (OR: 0.61, 95% CI, 0.39–0.94) was still associated with decreased rural career intent after controlling for gender, age, scholarship for regional duty, rural background, orientation towards general medicine, and intent to practice in a rural area. For Model B, the following variables were significant and dependent predictors for rural career intent after rural clinical training: rural self-efficacy (OR: 1.12, 95% CI, 1.07–1.16), scholarship for regional duty (OR: 1.71, 95% CI, 1.02–2.87), rural background (OR: 2.38, 95% CI, 1.31–4.34), orientation towards general medicine (OR: 1.87, 95% CI, 1.15–3.06), and intent to practice in a rural area (OR: 1.79, 95% CI, 1.09–2.93). These variables remained significant in the final model. Interestingly, the association between difficulty with living in a rural area and rural career intent disappeared after including rural self-efficacy (OR: 0.68, 95% CI, 0.43–1.06).

**Table 5:** Logistic regression analysis of baseline characteristics including rural self-efficacy on rural career intent after rural clinical training

**Baseline characteristics *N* = 563**	**Future rural career intent after rural clinical training**		
	**Non-adjusted**	**Multivariable-adjusted**	
	**OR (95% CI)**	**Model A** **OR (95% CI)**	**Model B** **OR (95% CI)**
Gender	0.89	0.77	0.90
Male vs. Female	(0.61–1.31)	(0.51–1.17)	(0.58–1.40)
Age	0.98	0.88	0.74
< 21 years vs. ≥ 21 years	(0.67–1.44)	(0.52–1.47)	(0.43–1.27)
Admission while living in hometown	1.41	1.10	1.08
Yes vs. No	(0.96**–**2.08)	(0.69–1.77)	(0.67–1.77)
Graduation from public high school	1.30	1.37	1.48
Yes vs. No	(0.89–1.92)	(0.63–2.96)	(0.67–3.28)
Graduation from junior high and high school	0.85	1.40	1.51
Yes vs. No	(0.58–1.25)	(0.65–2.99)	(0.69–3.31)
Has failed entrance exam	0.86	0.77	0.71
Yes vs. No	(0.58–1.27)	(0.44–1.33)	(0.40–1.25)
Work experience	1.22	1.13	0.94
Yes vs. No	(0.50–2.99)	(0.42–3.01)	(0.34–2.58)
Has a parent who is a doctor	0.81	0.88	0.88
Yes vs. No	(0.52–1.26)	(0.54–1.44)	(0.53–1.45)
Had a doctor as a role model	1.14	1.09	0.85
Yes vs. No	(0.77–1.68)	(0.71–1.68)	(0.54–1.33)
Scholarship for regional duty	**2.01**	**1.76**	**1.71**
Yes vs. No	**(1.30–3.11)**	**(1.07–2.91)**	**(1.02–2.87)**
Admission by school recommendation	1.13	0.81	0.73
Yes vs. No	(0.74–1.72)	(0.48–1.36)	(0.42–1.24)
Hometown of residence size until 18 years of age	**2.74**	**2.46**	**2.38**
Rural, remote, town, or village vs. small, medium, or large city	**(1.62–4.65)**	**(1.37–4.41)**	**(1.31–4.34)**
General medicine oriented	**2.25**	**2.18**	**1.87**
Yes vs. No	**(1.43–3.54)**	**(1.35–3.51)**	**(1.15–3.06)**
Degree of intent to practice in a rural area	**1.85**	**1.86**	**1.79**
Actively willing or for a certain period vs. avoid as much as possible, absolute refusal, or other	**(1.16–2.93)**	**(1.15–3.02)**	**(1.09–2.93)**
Difficulty with living in a rural area	**0.54**	**0.61**	0.68
Strongly agree or agree vs. disagree or strongly disagree	**(0.36–0.82)**	**(0.39–0.94)**	(0.43–1.06)
Rural self-efficacy	**1.12**		**1.12**
Per an increase in 1 score point	**(1.08–1.16)**		**(1.07–1.16)**

*OR* odds ratio, *CI* confidence interval. vs, versus. Model A, multivariable adjusted for all variables except for rural self-efficacy. Model B, multivariable adjusted for Model A + rural self-efficacy. Bold indicates significance (*p* < 0.05)

## Discussion

This study examined the relationship between baseline rural self-efficacy score and rural career intent after rural clinical training among medical students in Japan. The results showed that participants who found it more difficult to live in a rural area reported a more significant stepwise decrease in rural self-efficacy. Notably, rural self-efficacy showed a strong positive association with rural career intent even after rural clinical training, and this finding was independent of gender, scholarship for regional duty, rural background, orientation towards general medicine, baseline intent to practice in a rural area, and difficulty with living in a rural area. Thus, higher levels of self-efficacy could modulate the association between the reported difficulty in living in a rural area and rural career intent. To the best of our knowledge, Bandura’s [15] four sources of self-efficacy have not been used by previous questionnaires to measure the rural self-efficacy of medical students in Japan.

While numerous studies have examined the characteristics and identifiers of medical students to predict their intent to engage in rural practice, few explored factors affecting rural self-efficacy and the relationship between these factors and rural career intent. Often, studies highlighted rural origin [6, 25–27], having a spouse or significant other who lived in a rural area [26, 27], prior generalist intentions [6], and rural high school education [26] as factors influencing rural career intent. This study extends these findings by highlighting further factors significantly associated with rural career intent: rural self-efficacy and difficulty with living in a rural area. In Japan, the *chiiki-waku* scholarship for regional duty is a policy-based entrance program primarily aimed at increasing the number of doctors in rural areas. Our previous research showed that students who received *chiiki-waku* were positively motivated to engage in future rural practice [28]. Further, having a doctor as a role model, a rural background, and orientation towards general medicine were strongly associated with higher rural self-efficacy. However, rural self-efficacy remained significantly associated with rural career intent after rural clinical training, independent of these confounders. This study found that having a doctor as a role model was also associated with higher rural self-efficacy, although this association was not observed in the final model for rural career intent, possibly because the score item represented physicians as a role model [29].

Bentley et al. [20] reported higher rural self-efficacy scores for doctors in smaller towns (< 25,000 people) and small communities (< 10,000). Studies have also suggested that higher self-efficacy is associated with a rural background, a more senior career status, early decisions regarding specialization, and a smaller expectation–experience gap. This study included rural interprofessional work (RIPW) under students’ rural clinical training to examine rural career intent [30, 31]. Isaac et al. [11] suggested that the rural self-efficacy score may further improve after rural clinical training. Studies have also indicated that exposure to rural areas through education, recreation, and upbringing creates a sense of familiarity with the land, and community involvement motivates medical students to pursue intended or actual careers in rural areas [19, 32].

Self-efficacy is the feeling of being able to affirm one’s worth and existence [15]. Authors found that difficulty with living in a rural area was strongly associated with rural self-efficacy. However, the final model, which included rural self-efficacy, showed no such association between difficulty with living in a rural area and rural career intent. Participants with higher rural self-efficacy were likely to overcome difficulties associated with living in a rural area and report stronger rural career intent.

A key strength of this study was the follow-up data collection to examine the impact of rural efficacy on rural career intent both before and after rural training. Other strengths included the sample size and adjustment for possible confounding factors. However, this study had limitations. The study design did not eliminate potential causal relationships between the baseline characteristics of medical students and their rural practice intent. It was also limited to students from one regional university, so the results may not be generalizable. Finally, the study measured students’ intentions to practice in rural areas and not their actual practice choices.

## Conclusion

This study showed that higher self-efficacy strengthens the rural career intent of individuals after rural clinical training. Therefore, it is important to foster the development of rural self-efficacy to increase the recruitment and retention of general physicians in Japan’s rural communities. The impact of changes in rural self-efficacy before and after rural clinical training and their association with future rural career intent should also be examined.

## Data Availability

The survey data supporting this study’s conclusions are not publicly available to protect the confidentiality of participants, but are available from the corresponding author on reasonable request.

## Abbreviations

SD: Standard deviation
OR: Odds ratio
CI: Confidence interval
